# Relationship between Falls and the Use of Medications and Diseases in an Otago Exercise Programme in Old People Living in the Community in Spain

**DOI:** 10.3390/healthcare11070998

**Published:** 2023-03-31

**Authors:** Ana Covadonga González-Pisano, Maria Consuelo Company-Sancho, Eva Abad-Corpa, Maria Cristina Solé-Agusti, Maria Ángeles Cidoncha-Moreno, Marta M Pisano González

**Affiliations:** 1 Research Group “Community Health and Active Aging”, Biosanitary Research Institute of the Principality of Asturias, Primary Care, Ministry of Health, Asturias, 33006 Oviedo, Spain; 2 Health Promotion Service, Directorate General for Public Health, Canary Islands Health Service, 35003 Las Palmas de Gran Canaria, Spain; 3 Nursing and Healthcare Research Unit (Investén-Isciii), CIBER of Frailty and Healthy Aging (CIBER-FES), 30011 Murcia, Spain; 4 Murcia Health Service, Coordinator of the EIR AFYC Murcia East Program, 30100 Murcia, Spain; 5 Osakidetza-Basque Health Service, Subdirectorate of Nursing, 01006 Vitoria-Gasteiz, Spain; 6 General Direction of Care, Humanization and Social and Health Care, Principado de Asturias, Biosanitary Research Institute of the Principality of Asturias, Ministry of Health, 33006 Oviedo, Spain

**Keywords:** prevent falls, exercise, primary healthcare

## Abstract

(1) Background: Falls are a significant health problem among older adults, and can result in severe injuries, disability, and even death. In Spain, the prevalence of falls is lower if the person lives in the community than if they are institutionalized. Research has shown that exercise is an effective strategy for reducing the risk of falls among older adults. The objective of this study was to study the influence of a multicomponent exercise intervention on falls in people between 65 and 80 years of age despite the presence of diseases and drug use that are risk factors for falls; (2) Methods: This is a quasi-experimental study that focuses on people aged 65–80 who attended 21 primary healthcare centres. Target: Inclusion criteria were people between 65 and 80 years of age, living in the community with independent ambulation, and who were served by the healthcare centre of their region. Variables analysed: The number and characteristics of falls, sociodemographic, drug use, and previous diseases; (3) Results: The drugs associated with falls are benzodiazepines (OR 2.58), vasodilators (OR = 2.51), and psychotropics (OR = 1.61). For one of the years, a relationship was found between the consumption of antidepressants and falls (OR = 1.83). The associated diseases were mental and behavioural (OR = 2.53); (4) Discussion: The intervention has been related to the reduction in falls in people who consumed benzodiazepines, vasodilators, and psychotropics and in people with mental disorders; (5) Conclusion: This research concludes the importance of the implementation of the Otago Exercise Programme in the prevention of falls in the elderly.

## 1. Introduction

Falls are a major health problem due to their frequency and the problems derived from the impact. The World Health Organization (WHO) defines a fall as “an event which results in a person coming to rest inadvertently on the ground or floor or other lower level”, and which is usually sudden and involuntary [1].

Falls are very frequent and increase with age and frailty, as do their consequences, so much so that an estimated 646,000 people die from falls each year, and 37.3 million require medical assistance. They account for the loss of millions of disability-adjusted life years (DALYs) [2]. In Spain, the number of deaths due to falls in 2018 was 3143 [3]. The prevalence of falls is lower if the person lives in the community than if they are institutionalized. 

Within the associated morbidity and mortality are functional deterioration, loss of quality of life, increased dependency, visits to emergency services, hospital readmissions, and risk of institutionalization [3], among others. Meanwhile, falls are considered one of the most important geriatric syndromes and one of the indicators for identifying the frail elderly [4]. 

The population is ageing very quickly, with the WHO estimating that between 2015 and 2050 the population of over 60 s will increase from 900 million to 2 billion, which will account for 22% of the entire global population [2].

Spain has a rapidly ageing population and, as of 1 January 2020, 19.58% of the total population was 65 or over [5]. Within Spain, there are three regions that have an ageing index (number of elders per 100 persons younger than 15 years old in a specific population) greater than 200, Asturias (235.93), Castilla y León (231.84), and Galicia (212.37), which makes them the oldest regions in the country [6,7].

In addition to age, there are multiple risk factors associated with the risk of falls, such as the use of certain medications and the occurrence of diseases. 

The use of six or more medications (polypharmacy) is considered one of the most significant risk factors in the elderly population [8]. In a study on people who suffered falls, it was shown that the most influential medications were antidepressants, diuretics, sedatives, and antipsychotics [9]. Other authors highlight antihypertensives, followed by antidepressants and anxiolytics [10], or others, such as vasodilators, benzodiazepines, and neuroleptics [11].

A meta-analysis found significant results, the probability of falling being 1.3 times higher in people who consume diuretics [12] and 0.62 times higher in those who consume antihypertensives [13]. Another cohort study associates the consumption of psychotropic drugs with frailty and falls [14]. Consistent with this study, a meta-analysis confirmed that the consumption of psychotropic drugs was associated with a 1.54 times greater probability of falls, antidepressants with 1.57, and benzodiazepines with 1.42 [15].

Within the framework of the Health Promotion and Disease Prevention Strategy of the Spanish National Health System, the consensus document on the Prevention of Frailty and Falls in the Elderly [16] associates frailty with concomitant diseases such as neoplasms, autoimmune diseases, endocrine disorders, mental diseases, central nervous system diseases, heart diseases, digestive disorders, etc. Interventions based on physical exercise are proposed as part of the same strategy.

Falls, disability, and premature mortality are described as sequelae of musculoskeletal and mental diseases [17]. Likewise, some treatments for people with endocrine diseases increase the risk of hypocalcemia, falls, and fractures [18].

Given the strong correlation between mental illness and falls, this study provides detailed data on their prevalence in Spain. Anxiety disorders affect 6.7% of the population, followed by depressive disorders at 4.1%, sleep problems affect 5.4% and increase with age. Psychosis affects 7.2% of the population and dementia affects 3.2% of those over 60 years of age, as the main mental health problem. These data demonstrate the magnitude of mental health problems and, consequently, their relationship with falls [19].

Exercise is a subset of physical activity that is planned, structured, and repetitive, and has as an objective the improvement or maintenance of physical fitness [20]. There is a wide range of possible types of exercise, such as strengthening exercises, balance, coordination, and aerobic exercises. Exercise programmes that include one or more types of exercise are called multi-component exercise programmes. The inclusion of resistance training has been particularly effective in reversing frailty and preventing falls in older people [21]. 

Likewise, when relating multi-component exercise programmes with various previous diseases, a systematic review (SR) concludes that a multi-component exercise programme [22] reduces falls among the elderly. Other SRs corroborated the relationship of exercises to improve balance and reduce falls in cancer patients (Williams et al., 2018), after a cardiovascular accident [23], in people with rheumatoid arthritis [24] and in people with multiple sclerosis [25]. 

The target population of exercise programmes may exhibit a high use of medications [26] and may have illnesses [27]; therefore, it whether there may be a relationship between falls, medication use, and the occurrence of diseases among subjects participating in exercise programmes must be assessed. 

In this study we asked: Can a multicomponent exercise program reduce falls in a population that consumes medication or has diseases that increase their risk?

## 2. Materials and Methods

Design: this is a prospective and descriptive correlational multicentre study in non-institutionalized individuals aged between 65 and 80 years in Spain, randomly assigned, between September 2017 and December 2019. 

Scope: the participants were selected from primary care centres in ten Spanish provinces: Córdoba, Asturias, Balearic Islands, Las Palmas, Lérida, Barcelona, Madrid, Vitoria, Bilbao, and Murcia. 

Sample: Subjects who met the inclusion criteria and agreed to participate in a study of the Otago Exercise Programme (OEP). Inclusion criteria: individuals aged between 65 and 80 years, living in the community, with independent ambulation, and who attended to by the health centre in their region. Exclusion criteria: residency time in the health region less than 9 months, moderate or severe cognitive impairment, visual or hearing difficulties, or problems that contraindicated the performance of programme exercises; loss criteria were revocation of informed consent, change of residence, or changes in clinical status. Study interruption criteria: adverse effects related to the performance of Otago exercises

The sample size was calculated by estimating the percentage of annual falls among people over 65 years (40%), a Type I error of 0.025, a 1-βeta power equal to 0.80 in a unilateral contrast, and considering a 10% loss to follow-up, resulting in a sample of 364 participants. Active recruitment was carried out in health centres between the 10th and 19th (inclusive) of each month, from September 2017 to March 2018. 

Recruitment: The study participants were actively recruited by health professionals from health centres randomly selected from people who met the inclusion criteria. Recruitment was carried out consecutively in patients listed on the agenda between the 10th and 19th (inclusive) of each month. Each professional kept a notebook in which they recorded which patients had been proposed to, which ones met the criteria, and which ones agreed to participate.

OPE training: To standardize the intervention, the cascade training proposed by Later Life Training (Otago-LLT) and the online platform Otago-LLT for training in Spanish were used. In a first step, a professional from each participating subproject was accredited as a trainer of trainers (“Cascade Trainer”). In a second step, this accredited trainer trained health professionals (nurses and physiotherapists) in each participating health centre. The training and evaluation were standardized throughout Spain. After a positive evaluation, they were accredited as OPE Leaders.

These leaders also received standardized training on data collection and evaluation instruments or scales, ensuring validity and replicability.

Intervention: All participants included in this study followed the Otago Exercise Program (OEP) [28], either individually or in groups. It involves training participants in 24 strength and balance exercises, so they can continue the program at home. It is a 5-sessions training programme over 8 weeks, with a reinforcement booster session at 6 months. Each person received instructions for performing each prescribed exercise at home (in audiovisual and paper format), as well as weights for performing muscle-strengthening exercises.

Group sessions were only considered complete if the participant had completed all 5 sessions, and those who did not were recorded as lost in the analysis.

Variables:

The main response variable was the number and characteristics of each fall:

- Alone or accompanied;
- Consequences: none, minor or major temporary injury, minor or major permanent injury;

The secondary variables were:

- Sociodemographic: sex, age, level of education, and types of household;
- Anthropometric: body mass index (BMI);
- Medication use: psychotropics, diuretics, antidepressants, anticoagulants, antihypertensives, anticholinergics, and Vitamin D;
- Diseases: neoplastic, haematological, endocrinal, mental, central nervous system (CNS), sensory, cardiocirculatory, respiratory, digestive, dermatological, musculoskeletal, and/or genito-urinary.

For the data collection, a booklet was prepared, and the recruited subjects measured the variables at the beginning of the study, at 6 months (after the intervention) and at 1 year. The data were recorded on the online data platform REDCap. 

Statistical processing: The analyses were performed using the RStudio and IBM SPSS version 25 statistical packages, using descriptive statistics and measures of dispersion for quantitative variables.

It was verified that age and BMI (Kolmogorov–Smirnov test) did not present a normal distribution. 

Statistical inference: the Mann–Whitney test was used for quantitative variables. The Pearson’s Chi-square test was used to compare qualitative falls by sociodemographic characteristics and medications.

The risk was measured at the beginning of the study using odds ratio (at that time the fall had already happened), and the effect of the exposures was evaluated. For the follow-up at 6 and 12 months, the relative risk was measured (as the effects of medication on falls were known). 

A statistical significance was established for a *p*-value < 0.05.

This study was approved by the Research Ethics Committee of the Carlos III Health Institute with the CEI code PI_39_2016-v2.

## 3. Results

Out of a total of 2367 people who were interviewed, 827 met the inclusion criteria and signed the informed consent, and 498 participants of the OEP completed the intervention.

Table 1 shows the demographic and anthropometric data of the participants. 

The percentage of falls at baseline was 29.72%, at six months it was 11.85%, and on Year One it was 12.25%; therefore, the intervention with the OPE has proven to be effective after one year (*p* = 0.001).

Regarding the characteristics of the falls at baseline, the proportion of being alone at the time of the fall was significant (*p* = 0.011), with the percentage being 65.52% in women vs. 40.63% in men.

Regarding the need for healthcare after the fall, a significant relationship was observed (*p* = 0.039), with the percentage being 42.31% for women vs. 0% for men.

### 3.1. Use of Medication and Falls

The medications most used at baseline were antihypertensives 66.67% (including vasodilators and beta-blockers), followed by diuretics 34.14%, psychotropics 27.51% (including benzodiazepines, opiates, hypnotics, and neuroleptics), anticoagulants 17.87%, antidepressants 14.86%, and Vitamin D 12.05%.

On relating the medications to the presence of falls at baseline, the following was observed.

The use or non-use of psychotropic drugs exhibited significant differences in the proportion of falls, where the proportion of falls was higher in users of psychotropics compared to non-users, with patients who used psychotropic drugs being 1.61 times more likely to experience falls.

The proportion of falls was significantly higher in users of benzodiazepines compared to non-users, with patients who used benzodiazepines being 2.58 times more likely to experience falls.

Another significant finding was that the proportion of falls was higher in users of vasodilators compared to non-users, with patients who used vasodilators being 2.51 times more likely to experience falls.

The use or non-use of beta-blockers exhibited significant differences, where the proportion of falls was lower in users of beta-blockers compared to non-users, with patients who used beta-blockers being 0.46 (1–0.54) times less likely to experience falls in relation to non-users (Table 2).

At the end of the intervention at six months and relating the use of medications to the proportion of falls, no significant differences were observed, i.e., the risk factors observed at baseline for psychotropics, benzodiazepines, and vasodilators no longer existed after the intervention (Table 3).

When comparing the relationship between use of medications and the proportion of falls at twelve months post-intervention, significant differences were observed for antidepressants (*p* = 0.024), with the proportion of falls being 19.80% for users of this medication vs. 10.80% for non-users, with users of antidepressants having 1.83 times as many falls.

Table 4 shows the summary of the risks of the medications in terms of falls at the three points evaluated. At baseline, psychotropics, benzodiazepines, and vasodilators were risk factors for falls, while beta-blockers were a protective factor. At six months, the medications did not show a statistically significant relationship with falls, while in the post-intervention evaluation, it was observed that taking antidepressants was a risk factor.

### 3.2. Diseases and Falls

In the baseline analysis of previous diseases, 69.68% were cardiocirculatory, 51.40% musculoskeletal, 43.78% endocrinal, 23.49% digestive, 23.29% genito-urinary, 14.06% sensory, 13.86% respiratory, 12.65% dermatological, and 12.45% neoplastic.

On relating them to the presence of falls at baseline, significant differences were observed for mental and behavioural diseases (*p* = 0.001), where the proportion of falls was 49% for patients with these illnesses and 27.50% for patients without these illnesses. Patients with mental and behavioural disorders were 2.53 times more likely to experience falls than patients without these diseases.

Musculoskeletal diseases and the presence of falls at baseline exhibited a *p*-value of 0.052, which is approximately equal to the level of significance (0.05), and obtained clinical significance given that the proportions of falls were 33.60% for patients with these diseases vs. 25.60% for patients without these diseases (Table 5).

The relationship between the proportion of falls at 6 months and the clinical presentation exhibited significance for musculoskeletal disease. On comparing the proportion of one year post-intervention falls on Year One according to the clinical presentation, significance was observed for musculoskeletal disease (*p* = 0.007), where the proportion of falls was 15.87% for patients with this disease vs. 7.93% for patients without this disease, with patients with musculoskeletal disease being twice as likely to experience falls (Table 6).

Table 7 shows the summary of the clinical risks of falls at the three times they were evaluated; for the baseline, mental and behavioural disorders OR = 2.53 (1.41–4.56) were a risk factor for falls. The end-of-intervention (6 months) and post-intervention (7 to 12 months) evaluations showed musculoskeletal disease to be a risk factor for falls with OR 1.72 (1.03–2.85) and 2.00 (1.19–3.37), respectively.

## 4. Discussion

This study explored the relationship between an Otago Exercise Programme and the use of medications and the diseases of the participating subjects.

At baseline, the medications that were associated with falls were, in this order, benzodiazepines (highlighted because of their strong association), vasodilators, and psychotropics. On Year One, an association was found between the use of antidepressants and falls.

At the end of the intervention and on Year One, these risk factors (use of benzodiazepines, vasodilators, and psychotropics) observed at the beginning were no longer risk factors. This may be due to the significant decrease in the proportion of falls from 29.72% at the beginning to 11.85%.

These three medications have also been identified and associated with falls in numerous studies [9,10,29,30].

It should be mentioned that other studies have found associations with other medications such as diuretics [9,26] and antihypertensives [8,10]

The association of vasodilators with falls could be explained by the effect they produce by lowering blood pressure, as could the association with antihypertensives [31]. However, we have not found the reason why beta-blocking drugs provide a protective effect and Vitamin D does not, as other studies indicate [32].

Studies confirm the benefits of multi-component exercise programmes to improve antihypertensive treatment and cardiovascular risk in older adults with comorbidities. [33].

Our results partly agree with another study, where the medications most associated with the presence of falls were antidepressants, diuretics, sedatives, and antipsychotics [9], and with the study by Fabra, where it was antihypertensives followed by antidepressants and anxiolytics [10].

Previous research has shown a significant relationship between antidepressants, diuretics, and falls [18], and among antidepressants, psychotropic medication appears to be the most significant risk factor [34].

Another study found that more women than men who presented for emergency care after a fall were prescribed benzodiazepines, but men were prescribed significantly higher doses than women [30]. Another systematic review suggests a relationship between the use of benzodiazepines, falls, and gender differences [35].

In our study, the prevalence of falls decreased after intervention in people who consumed psychotropic drugs. This is a significant finding. There are studies that show an improvement in symptoms of mental disorders when a physical exercise program is combined with pharmacological treatment, which could have an impact on improving physical condition [36].

There are even studies that suggest that treatments based on exercise change structures of the central nervous system, with benefits for mental diseases [37]. More studies have examined the efficacy of regular physical exercise programs in the treatment and improvement of mental problems [38], which could suggest that the decrease in falls in people who consumed psychotropics is due to improved physical condition (strengthening and balance) and the benefits of exercise for their disease.

Other studies found that the use of antihypertensives was related to hip fractures [8]; antidepressants and psychotropics were related with falls and hospitalizations [39], and hypnotics, sedatives and opiates highlight the impact on women [40].

Regarding previous diseases, this study found that having a mental disorder was significantly associated with a 2.53 times greater risk of suffering a fall at baseline. This risk disappeared after the intervention and was maintained over time, so the OEP could provide a protective effect for people suffering from these disorders.

Our results on the relationship between mental diseases and falls are consistent with those of other studies [41,42].

In this study, people with musculoskeletal disease had a risk 1.72 times greater at six months of suffering a fall, and 2 times greater at one year than those without the disease. This risk is associated with the deterioration of the joints, mainly of the lower extremities, which causes problems with instability, balance, coordination, and muscular strength. It is unknown why the OEP increased the risk of falls among these patients. Moreover, an SR on balance training in patients with rheumatoid arthritis did not obtain conclusive data [24].

Thanks to current treatments, the survival rates of cancer patients has increased, and the rates of falls in cancer patients or cancer survivors are higher than in the general population. A current SR suggests that an exercise programme can improve strength, flexibility, and balance, although it recommends performing quality studies to gain more evidence [43]. No significant relationships were found in this study.

Neither were any relationships found between endocrinal diseases and falls, unlike a study carried out on 1422 patients, which found that diabetes mellitus increased gait and balance problems by 1.25 [44].

Multiple sclerosis is one of the most prevalent CNS diseases, with rates of falls at around 56%. Although the evidence on the decrease in falls with exercise programmes is low, they can improve balance and functionality [25]. In this study, no significant differences were found with respect to CNS and sensory diseases.

With regard to cardiovascular diseases, the sensory organs, dermatological, and genito-urinary diseases, our data are inconsistent with other studies, such as those that found relationships between falls and stroke survivors [23], people with vision and hearing problems [8], and people who had chronic kidney disease [45].

Multi-component exercises can be beneficial to functional status and cardiovascular risk in patients with hypertension [46], and in older people who are frail or have multiple diseases [47].

The practical and theoretical implications of our research suggest that the systematic implementation of a fall prevention exercise program in health centres could significantly benefit elderly individuals. We recommend that further studies are conducted in older or frail groups to determine whether the benefits could be even greater.

In summary, our study highlights the importance of exercise programs as a preventive measure for falls in the elderly population, particularly those who consume psychotropic drugs. Implementing exercise programs in health centres could help reduce the prevalence of falls and improve physical condition, thereby reducing the negative impact of falls on the elderly.

As a limitation of this study and a proposal for future research could be the fact that multimorbidity may have a different effect on falls if the associated diseases are studied, instead of studying each disease in isolation. Perhaps this is the point where we can find the difference found in this study with respect to others in terms of the relationship of previous diseases with falls.

## 5. Conclusions

The OPE program intervention has been proven to be effective in reducing falls in the community-dwelling population between 65 and 80 years of age. The exercise program’s greatest benefits were found in women and men over the age of 72 and people who live alone.

At baseline, medications related to falls included benzodiazepines, vasodilators, and psychotropics. At the one-year follow-up, only the relationship between antidepressants and falls remained statistically significant.

Mental and behavioural disorders were significantly associated with falls only at baseline, with no significant relationship found at the six months post-test and one-year follow-up.

The intervention was associated with a decrease in falls in people who used benzodiazepines, vasodilators, and psychotropics, as well as in people with mental disorders.

Our results suggest that controlling for medications that are potentially related to falls in older adults may help improve exercise intervention designs and ultimately increase their treatment effects.

## Figures and Tables

**Table 1:** Sociodemographic and anthropometric characteristics.

Sociodemographic and Anthropometric Characteristics	Results
Age (average (SD)) ^1/^ years	71.81 (4.15)
Sex (n (%)) ^2/^	
Females	334 (67.07)
Male	164 (32.93)
Educational level (n (%)) ^2/^	
Without studies	43 (8.63)
Primary non-concluded	106 (21.29)
Primary concluded	226 (45.38)
Secondary	82 (16.47)
University studies	41 (8.23)
Living alone (n (%))^2/^	
Yes	122 (24.5)
Not	376 (75.5)
BIM (average (DE)) ^1/^	29.36 (4.49)
BIM clasification (n (%)) ^2/^	
Under-Weight	1 (0.2)
Normal	108 (21.69)
Over-Weight	186 (37.35)
Obesity	196 (39.36)
Extreme Obesity	7 (1.41)

Note: SD = standard deviation. BIM: body mass index. ^2/^: based on the Chi-square test

**Table 2:** Relationship between falls at baseline and medications.

Medications (Baseline)	General	Fall at Baseline (Last 12 Months)		*p*-Value	OR^2^ (CI-95%)
		Yes	No		
Psychotropics (n (%))					
Yes	137 (27.51)	51 (37.23)	86 (62.77)	0.024 *	1.61 ** (1.06–2.45)
No	361 (72.49)	97 (26.87)	264 (73.13)		
Benzodiazepines (n (%))					
Yes	105 (76.64)	44 (41.9)	61 (58.1)	0.040 *	2.58 ** (1.03–6.49)
No	32 (23.36)	7 (21.88)	25 (78.13)		
Opiates (n (%))					
Yes	24 (17.52)	11 (45.83)	13 (54.17)	0.337	1.54 (0.63–3.76)
No	113 (82.48)	40 (35.4)	73 (64.6)		
Hypnotics (n (%))					
Yes	13 (9.49)	2 (15.38)	11 (84.62)	0.087	0.28 (0.06–1.31)
No	124 (90.51)	49 (39.52)	75 (60.48)		
Diuretics (n (%))					
Yes	170 (34.14)	53 (31.18)	117 (68.82)	0.608	1.11 (0.74–1.66)
No	328 (65.86)	95 (28.96)	233 (71.04)		
Antidepressants (n (%))					
Yes	74 (14.86)	28 (37.84)	46 (62.16)	0.098	1.54 (0.92–2.58)
No	424 (85.14)	120 (28.3)	304 (71.7)		
Anticoagulants (n (%))					
Yes	89 (17.87)	22 (24.72)	67 (75.28)	0.255	0.74 (0.44–1.25)
No	409 (82.13)	126 (30.81)	283 (69.19)		
Antihypertensives (n (%))					
Yes	332 (66.67)	100 (30.12)	232 (69.88)	0.782	1.06 (0.70–1.60)
No	166 (33.33)	48 (28.92)	118 (71.08)		
Vasodilators (n (%))					
Yes	294 (88.55)	94 (31.97)	200 (68.03)	0.041 *	2.51 ** (1.01–6.02)
No	38 (11.45)	6 (15.79)	32 (84.21)		
Beta-blockers (n (%))					
Yes	98 (29.52)	21 (21.43)	77 (78.57)	0.025 *	0.54 *** (0.31–0.93)
No	234 (70.48)	79 (33.76)	155 (66.24)		
Vitamin D (n (%))					
Yes	60 (12.05)	20 (33.33)	40 (66.67)	0.514	1.21 (0.68–2.15)
No	438 (87.95)	128 (29.22)	310 (70.78)		

Note: * significant differences in the proportion of falls, OR^2^: based on the Chi-square test; significant odds ratio (OR) ** risk factor, *** protective factor.

**Table 3:** Relationship between falls in the six-month follow-up and medications.

Medications (6 Months)	General	6-Month Follow-Up after Fall		*p*-Value	RR (CI-95%)
		Yes	No		
Psychotropics (n (%))					
Yes	140 (28.11)	18 (12.86)	122 (87.14)	0.663	1.12 (0.67–1.89)
No	358 (71.89)	41 (11.45)	317 (88.55)		
Benzodiazepines (n (%))					
Yes	106 (75.71)	16 (15.09)	90 (84.91)	0.241	2.56 (0.62–10.60)
No	34 (24.29)	2 (5.88)	32 (94.12)		
Opiates (n (%))					
Yes	22 (15.71)	1 (4.55)	21 (95.45)	0.306	0.32 (0.04–2.25)
No	118 (84.29)	17 (14.41)	101 (85.59)		
Neuroleptics (n (%))					
Yes	8 (5.71)	0 (0)	8 (100)	0.596	-
No	132 (94.29)	18 (13.64)	114 (86.36)		
Hypnotics (n (%))					
Yes	22 (15.71)	1 (4.55)	21 (95.45)	0.306	0.32 (0.04–2.25)
No	118 (84.29)	17 (14.41)	101 (85.59)		
Diuretics (n (%))					
Yes	161 (32.33)	19 (11.8)	142 (88.2)	0.982	0.99 (0.60–1.66)
No	337 (67.67)	40 (11.87)	297 (88.13)		
Antidepressants (n (%))					
Yes	80 (16.06)	9 (11.25)	71 (88.75)	0.857	0.93 (0.44–1.98)
No	418 (83.94)	50 (11.96)	368 (88.04)		
Anticoagulants (n (%))					
Yes	92 (18.47)	8 (8.7)	84 (91.3)	0.300	0.69 (0.34–1.41)
No	406 (81.53)	51 (12.56)	355 (87.44)		
Antihypertensives (n (%))					
Yes	340 (68.27)	40 (11.76)	300 (88.24)	0.933	0.98 (0.59–1.63)
No	158 (31.73)	19 (12.03)	139 (87.97)		
Vasodilators (n (%))					
Yes	308 (90.59)	33 (10.71)	275 (89.29)	0.080	0.49 (0.24–1.02)
No	32 (9.41)	7 (21.88)	25 (78.13)		
Beta-blockers (n (%))					
Yes	99 (29.03)	12 (12.12)	87 (87.88)	0.886	1.05 (0.55–1.976)
No	242 (70.97)	28 (11.57)	214 (88.43)		
Vitamin D (n (%))					
Yes	59 (11.85)	7 (11.86)	52 (88.14)	0.997	1.00 (0.48–2.10)
No	439 (88.15)	52 (11.85)	387 (88.15)		

Note: based on Chi-square test; relative risk (RR).

**Table 4:** Relationship between falls and use of medications.

Medications	Risk		
	Baseline	Follow-Up	
		6 Months	7 to 12 Months
	OR (CI-95%)	RR (CI-95%)	RR (CI-95%)
Psychotropics	1.61 ** (1.06–2.45)	1.12 (0.67–1.89)	1.37 (0.83–2.24)
Benzodiazepines	2.58 ** (1.03–6.49)	2.56 (0.62–10.60)	1.18 (0.46–3.02)
Opiates	1.54 (0.63–3.76)	0.32 (0.04–2.25)	1.73 (0.74–4.07)
Hypnotics	0.28 (0.06–1.31)	0.32 (0.04–2.25)	1.12 (0.41–3.04)
Diuretics	1.11 (0.74–1.66)	0.99 (0.60–1.66)	0.93 (0.56–1.55)
Antidepressants	1.54 (0.92–2.58)	0.93 (0.44–1.98)	1.83 ** (1.10–3.07)
Anticoagulants	0.74 (0.44–1.25)	0.69 (0.34–1.41)	1.13 (0.63–2.03)
Antihypertensives	1.06 (0.70–1.60)	0.98 (0.59–1.63)	0.85 (0.52–1.38)
Vasodilators	2.51 ** (1.01–6.02)	0.49 (0.24–1.02)	0.95 (0.36–2.51)
Beta-blockers	0.54 *** (0.31–0.93)	1.05 (0.55–1.976)	0.97 (0.51–1.87)
Vitamin D	1.21 (0.68–2.15)	1.00 (0.48–2.10)	0.93 (0.44–1.95)

Note: ** risk factor; *** protective factor.

**Table 5:** Relationship between falls at baseline and clinical presentation.

Clinical Presentation (Baseline)	General	Fall at Baseline (Last 12 Months)		*p*-Value	OR^2^ (CI-95%)
		Yes	No		
Neoplastic (n (%))					
Yes	62 (12.45)	21 (33.87)	41 (66.13)	0.445	1.25 (0.71–2.19)
No	436 (87.55)	127 (29.13)	309 (70.87)		
Immune and haematological (n (%))					
Yes	36 (7.23)	10 (27.78)	26 (72.22)	0.791	0.90 (0.42–1.92)
No	462 (92.77)	138 (29.87)	324 (70.13)		
Endocrinal (n (%))					
Yes	218 (43.78)	59 (27.06)	159 (72.94)	0.253	0.80 (0.54–1.18)
No	280 (56.22)	89 (31.79)	191 (68.21)		
Mental and behavioural (n (%))					
Yes	51 (10.2)	25 (49)	26 (51)	0.001 *	2.53 ** (1.41–4.56)
No	447 (89.8)	123 (27.5)	324 (72.5)		
CNS and sensory (n (%))					
Yes	52 (10.44)	13 (25)	39 (75)	0.431	0.77 (0.40–1.49)
No	446 (89.56)	135 (30.27)	311 (69.73)		
Cardiocirculatory (n (%))					
Yes	347 (69.68)	104 (29.97)	243 (70.03)	0.852	1.04 (0.68–1.58)
No	151 (30.32)	44 (29.14)	107 (70.86)		
Sensory (n (%))					
Yes	70 (14.06)	18 (25.71)	52 (74.29)	0.429	0.79 (0.48–1.41)
No	428 (85.94)	130 (30.37)	298 (69.63)		
Respiratory (n (%))					
Yes	69 (13.86)	21 (30.43)	48 (69.57)	0.889	1.04 (0.60–1.81)
No	429 (86.14)	127 (29.6)	302 (70.4)		
Digestive (n (%))					
Yes	117 (23.49)	35 (29.91)	82 (70.09)	0.958	1.01 (0.64–1.59)
No	381 (76.51)	113 (29.66)	268 (70.34)		
Dermatological (n (%))					
Yes	63 (12.65)	20 (31.75)	43 (68.25)	0.706	1.12 (0.63–1.97)
No	435 (87.35)	128 (29.43)	307 (70.57)		
Musculoskeletal (n (%))					
Yes	256 (51.4)	86 (33.6)	170 (66.4)	0.052	1.47 (0.99–2.17)
No	242 (48.6)	62 (25.6)	180 (74.4)		
Genito-urinary (n (%))					
Yes	116 (23.29)	31 (26.72)	85 (73.28)	0.420	0.83 (0.52–1.32)
No	382 (76.71)	117 (30.63)	265 (69.37)		

Note: * significant differences in the proportion of falls, OR^2^: based on the Chi-square test; significant odds ratio (OR) ** risk factor; CNS: central nervous system.

**Table 6:** Relationship between falls at 12 months and clinical presentation.

Clinical Presentation (12 Months)	General	Post-Intervention Fall 12 Months		*p*-Value	OR^2^ (CI-95%)
		Yes	No		
Neoplastic (n (%))					
Yes	60 (12.05)	11 (18.33)	49 (81.67)	0.125	1.61 (0.87–2.91)
No	438 (87.95)	50 (11.42)	388 (88.58)		
Immune and haematological (n (%))					
Yes	40 (8.03)	5 (12.5)	35 (87.5)	1.000	1.02 (0.43–2.41)
No	458 (91.97)	56 (12.23)	402 (87.77)		
Endocrinal (n (%))					
Yes	221 (44.38)	26 (11.76)	195 (88.24)	0.768	0.93 (0.58–1.50)
No	277 (55.62)	35 (12.64)	242 (87.36)		
Mental and behavioural (n (%))					
Yes	54 (10.84)	8 (14.81)	46 (85.19)	0.542	1.24 (0.62–2.47)
No	444 (89.16)	53 (11.94)	391 (88.06)		
CNS and sensory (n (%))					
Yes	45 (9.04)	3 (6.67)	42 (93.33)	0.231	0.52 (0.17–1.60)
No	453 (90.96)	58 (12.8)	395 (87.2)		
Cardiocirculatory (n (%))					
Yes	355 (71.29)	41 (11.55)	314 (88.45)	0.453	0.83 (0.50–1.36)
No	143 (28.71)	20 (13.99)	123 (86.01)		
Sensory (n (%))					
Yes	73 (14.66)	9 (12.33)	64 (87.67)	0.982	1.01 (0.52–1.96)
No	425 (85.34)	52 (12.24)	373 (87.76)		
Respiratory (n (%))					
Yes	76 (15.26)	11 (14.47)	65 (85.53)	0.520	1.22 (0.67–2.24)
No	422 (84.74)	50 (11.85)	372 (88.15)		
Digestive (n (%))					
Yes	121 (24.3)	16 (13.22)	105 (86.78)	0.707	1.11 (0.65–1.89)
No	377 (75.7)	45 (11.94)	332 (88.06)		
Dermatological (n (%))					
Yes	72 (14.46)	7 (9.72)	65 (90.28)	0.480	0.77 0.36–1.62)
No	426 (85.54)	54 (12.68)	372 (87.32)		
Musculoskeletal (n (%))					
Yes	271 (54.42)	43 (15.87)	228 (84.13)	0.007 *	2.00 ** (1.19–3.37)
No	227 (45.58)	18 (7.93)	209 (92.07)		
Genito-urinary (n (%))					
Yes	118 (23.69)	11 (9.32)	107 (90.68)	0.267	0.71 (0.38–1.32)
No	380 (76.31)	50 (13.16)	330 (86.84)		

Note: * significant differences in the proportion of falls, OR^2^: based on the Chi-square test; significant odds ratio (OR) ** risk factor. CNS: central nervous system.

**Table 7:** Relationship between falls and previous diseases.

Clinical Presentation (Baseline)	Risk		
	Baseline	Follow-Up	
		6 Months	7 to 12 Months
	OR (CI-95%)	RR (CI-95%)	RR (CI-95%)
Neoplastic	1.25 (0.71–2.19)	0.69 (0.28–1.62)	1.61 (0.87–2.91)
Immune and haematological	0.90 (0.42–1.92)	0.43 (0.11–1.67)	1.02 (0.43–2.41)
Endocrinal	0.80 (0.54–1.18)	1.07 (0.66–1.72)	0.93 (0.58–1.50)
Mental and behavioural	2.53 ** (1.41–4.56)	0.79 (0.33–1.90)	1.24 (0.62–2.47)
CNS and sensory	0.77 (0.40–1.49)	0.91 (0.38–2.16)	0.52 (0.17–1.60)
Cardiocirculatory	1.04 (0.68–1.58)	0.91 (0.54–1.51)	0.83 (0.50–1.36)
Sensory	0.79 (0.48–1.41)	0.94 (0.47–1.90)	1.01 (0.52–1.96)
Respiratory	1.04 (0.60–1.81)	1.70 (0.97–2.98)	1.22 (0.67–2.24)
Digestive	1.01 (0.64–1.59)	0.86 (0.48–1.54)	1.11 (0.65–1.89)
Dermatological	1.12 (0.63–1.97)	0.99 (0.49–1.99)	0.77 0.36–1.62)
Musculoskeletal	1.47 (0.99–2.17)	1.72 ** (1.03–2.85)	2.00 ** (1.19–3.37)
Genito-urinary	0.83 (0.52–1.32)	0.79 (0.43–1.43)	0.71 (0.38–1.32)

Note: **risk factor. CNS: central nervous system.

## Data Availability

Not applicable.

## References

[1] World Health Organization 71.a ASAMBLEA MUNDIAL DE LA SALUD A71/4. Punto 11.1 del Orden del día Provisional 5 de Abril de 2018. Informe del Director General. https://apps.who.int/gb/ebwha/pdf_files/WHA71/A71_4-sp.pdf.

[2] World Health Organization OMS|Enfermedades no Transmisibles. https://www.who.int/topics/noncommunicable_diseases/es/.

[3] Rodríguez-Molinero A., Narvaiza L., Gálvez-Barrón C., de la Cruz J.J., Ruíz J., Gonzalo N., Valldosera E., Yuste A. (2015). Caídas en la población anciana española: Incidencia, consecuencias y factores de riesgo. Rev. Esp. Geriatría Gerontol..

[4] González Ramírez A., Calvo Aguirre J.J., Lekuona Ancizar P., González Oliveras J.L., Marcellán Benavente T., Ruiz de Gordoa Armendia A., Salvá Casanovas A., Alcalde Tirado P., González Alonso T., Padilla Clemente R. (2013). El fenómeno de las caídas en residencias e instituciones: Revisión del Grupo de Trabajo de Osteoporosis, Caídas y Fracturas de la Sociedad Española de Geriatría y Gerontología (GCOF-SEGG). Rev. Esp. Geriatría Gerontol..

[5] Pérez-Díaz J., Abellan-García A., Aceituno-Nieto P., Ramiro-Fariñas D. Un Perfil de las Personas Mayores en España, 2020. Indicadores Estadísticos Básicos. Informes Envejecimiento en red no 25. http://envejecimiento.csic.es/documentos/documentos/enred-indicadoresbasicos2020.pdf.

[6] Instituto Nacional de Estadística INE. https://www.ine.es/dyngs/INEbase/es/operacion.htm?c=Estadistica_C&cid=1254736176951&menu=ultiDatos&idp=1254735572981.

[7] Preedy V.R., Watson R.R. (2010). Handbook of Disease Burdens and Quality of Life Measures.

[8] Vidal M., Lisbeth C. La Polifarmacia Como Factor de Riesgo Para Fractura de Cadera en Adultos Mayores. Hospital Regional Docente de Trujillo, periodo 2017–2018. http://dspace.unitru.edu.pe/handle/UNITRU/14118.

[9] Carballo-Rodríguez A., Gómez-Salgado J., Casado-Verdejo I., Ordás B., Fernández D., Carballo-Rodríguez A., Gómez-Salgado J., Casado-Verdejo I., Ordás B., Fernández D. (2018). Estudio de Prevalencia y Perfil de Caídas En Ancianos Institucionalizados. Gerokomos.

[10] Varas-Fabra F., Castro Martín E., Pérula de Torres L.Á., Fernández Fernández M.J., Ruiz Moral R., Enciso Berge I. (2006). Caídas en ancianos de la comunidad: Prevalencia, consecuencias y factores asociados. Aten. Primaria.

[11] Díaz Grávalos G.J., Gil Vázquez C., Andrade Pereira V., Alonso Payo R., Álvarez Araujo S., Reinoso Hermida S. (2009). Factores asociados con la aparición de caídas en ancianos institucionalizados: Un estudio de cohortes. Rev. Esp. GeriatrÍa Gerontol..

[12] De Vries N.M., Seppala L.J., Daams J.G., Van-de-Glind E., Masud T., Van-der-Velde N. (2018). EUGMS Task and Finish Group on Fall-Risk-Increasing Drugs. Fall-Risk-Increasing Drugs: A Systematic Review and Meta-Analysis: I. Cardiovascular Drugs. J. Am. Med. Dir. Assoc..

[13] Lipsitz L., Habtemariam D., Gagnon M., Iloputaife I., Sorond F., Tchalla A., Dantoine T., Travison T. (2015). Reexamining the Effect of Antihypertensive Medications on Falls in Old Age. Hypertension.

[14] Aprahamian I., Landowski A., Ahn F.O., Neves B.A., Rocha J.T., Strauss J., Borges M.K., Morley J.E., Voshaar R.C.O. (2022). Frailty in geriatric psychiatry inpatients: A retrospective cohort study. Int. Psychogeriatr..

[15] Seppala L., Wermelink A., De Vries M., Ploegmakers K., Van de Glind E., Daams J. (2018). Fall-Risk-Increasing Drugs: A Systematic Review and Meta-Analysis: II. Psychotropics. J. Am. Med. Dir. Assoc..

[16] Ministerio de Sanidad, Servicio Sociales e Igualdad Documento de Consenso Sobre Prevención y Caídas en la Persona Mayor. https://www.mscbs.gob.es/profesionales/saludPublica/prevPromocion/Estrategia/docs/Fragilidad/FragilidadyCaidas_personamayor.pdf.

[17] Vaughan L., Corbin A., Goveas J. (2015). Depression and frailty in later life: A systematic review. Clin. Interv. Aging.

[18] Espeland M.A., Pratley R.E., Rosenstock J., Kadowaki T., Seino Y., Zinman B., Marx N., McGuire D.K., Andersen K.R., Mattheus M. (2021). Cardiovascular outcomes and safety with linagliptin, La dipeptidyl peptidase-4 inhibitor, compared with the sulphonylurea glimepiride in older people with type 2 diabetes: A subgroup analysis of the randomized CAROLINA trial. Diabetes Obes. Metab..

[19] García F.M., Calvo-Reyes M.C., Rodríguez-Cobo I. Salud Mental en Datos: Prevalencia de los Problemas de Salud y Consumo de Psicofármacos y Fármacos Relacionados a Partir de Registros Clínicos de Atención Primaria. Sistema Nacional de Salud. Ministerio de Sanidad. https://www.sanidad.gob.es/estadEstudios/estadisticas/estadisticas/estMinisterio/SIAP/Salud_mental_datos.pdf.

[20] Caspersen C.J., Powell K.E., Christenson G.M. (1985). Physical activity, exercise, and physical fitness: Definitions and distinctions for health-related research. Public Health Rep..

[21] Cadore E.L., Rodríguez-Mañas L., Sinclair A., Izquierdo M. (2013). Effects of Different Exercise Interventions on Risk of Falls, Gait Ability, and Balance in Physically Frail Older Adults: A Systematic Review. Rejuvenation Res..

[22] Sherrington C., Fairhall N.J., Wallbank G.K., Tiedemann A., Michaleff Z.A., Howard K., Clemson L., Hopewell S., Lamb S.E. (2019). Exercise for Preventing Falls in Older People Living in the Community. Cochrane Database Syst. Rev..

[23] Denissen S., Staring W., Kunkel D., Pickering R.M., Lennon S., Geurts A.C., Weerdesteyn V., Verheyden G.S. (2019). Interventions for Preventing Falls in People after Stroke. Cochrane Database Syst. Rev..

[24] Silva K.N., Imoto A.M., Almeida G.J., Atallah Á.N., Peccin M.S., Trevisani V.F.M. (2010). Balance Training (Proprioceptive Training) for Patients with Rheumatoid Arthritis. Cochrane Database Syst. Rev..

[25] Hayes S., Galvin R., Kennedy C., Finlayson M., McGuigan C., Walsh C.D., Coote S. (2019). Interventions for Preventing Falls in People with Multiple Sclerosis. Cochrane Database Syst. Rev..

[26] Valenzuela O.C., García I.R., Saldaña A.T. (2020). Factores de riesgo para síndrome de caídas en adultos mayores con polifarmacia. At. Fam..

[27] Albornos-Muñoz L., Moreno-Casbas M.T., Sánchez-Pablo C., Bays-Moneo A., Fernández-Domínguez J.C., Rich-Ruiz M., Gea-Sánchez M., The Otago Project Working Group (2018). Efficacy of the Otago Exercise Programme to reduce falls in community-dwelling adults aged 65–80 years old when delivered as group or individual training. J. Adv. Nurs..

[28] Goto N.A., Hamaker M.E., Willems H.C., Verhaar M.C., Emmelot-Vonk M.H. (2019). Accidental Falling in Community-Dwelling Elderly with Chronic Kidney Disease. Int. Urol. Nephrol..

[29] Gama Z.A.d.S., Gómez-Conesa A. (2008). Factores de riesgo de caídas en ancianos: Revisión sistemática. Rev. Saúde Pública.

[30] Martinez-Cengotitabengoa M., Diaz-Gutierrez M.J., Besga A., Bermúdez-Ampudia C., López P., Rondon M.B., Stewart D.E., Perez P., Gutierrez M., Gonzalez-Pinto A. (2018). Prescripción de benzodiacepinas y caídas en mujeres y hombres ancianos. Rev. Psiquiatr. Salud Ment..

[31] Deng A., Conrad K., Baylis C. (2018). Relaxin-mediated renal vasodilation in the rat is associated with falls in glomerular blood pressure. Am. J. Physiol.-Regul. Integr. Comp. Physiol..

[32] Fuentes-Barría H., Aguilera-Eguia R., González-Wong C., Fuentes-Barría H., Aguilera-Eguia R., González-Wong C. (2018). The Role of Vitamin D in the Prevention of Falls among Subjects with Sarcopenia. Rev. Chil. Nutr..

[33] Baptista L.C., Machado-Rodrigues A.M., Veríssimo M.T., Martins R.A. (2018). Exercise Training Improves Functional Status in Hypertensive Older Adults under Angiotensin Converting Enzymes Inhibitors Medication. Exp. Gerontol..

[34] Díaz M.J. (2020). Sobredosificación de Benzodiazepinas en Ancianos y Caídas: Implicaciones Clínicas y Económicas. Doctor’s Thesis.

[35] Sun M., Min L., Xu N., Huang L., Li X. (2021). The Effect of Exercise Intervention on Reducing the Fall Risk in Older Adults: A Meta-Analysis of Randomized Controlled Trials. Int. J. Environ. Res. Public Health.

[36] Belvederi-Murri M., Amore M., Menchetti M., Toni G., Neviani F., Cerri M. (2015). Safety and Efficacy of Exercise for Depression in Seniors (SEEDS) Study Group. Physical exercise for late-life major depression. Br. J. Psychiatry.

[37] Gultyaeva V.V., Zinchenko M.I., Uryumtsev D.Y., Krivoschekov S.G., Aftanas L.I. (2019). Fizicheskaia nagruzka pri lechenii depressii. Fiziologicheskie mekhanizmy [Exercise for depression treatment. Physiological mechanisms]. S.S. Korsakov J. Neurol. Psychiatry.

[38] Kok R.M., Reynolds C.F. (2017). Management of Depression in Older Adults: A Review. JAMA.

[39] Johnell K., Jonasdottir Bergman G., Fastbom J., Danielsson B., Borg N., Salmi P. (2017). Psychotropic Drugs and the Risk of Fall Injuries, Hospitalisations and Mortality among Older Adults. Int. J. Geriatr. Psychiatry.

[40] Fernández M., Valbuena C., Natal C. (2018). Riesgo de caídas asociado al consumo de medicamentos en la población anciana. J. Healthc. Qual. Res..

[41] Wei T.-S., Liu P.-T., Chang L.-W., Liu S.-Y. (2017). Gait asymmetry, ankle spasticity, and depression as independent predictors of falls in ambulatory stroke patients. PLoS ONE.

[42] Casahuaman-Orellana L., Runzer-Colmenares F.M., Parodi J.F. (2019). Asociación entre síndrome de caídas y síntomas depresivos en adultos mayores de once comunidades altoandinas del Perú 2013–2017. Rev. Neuropsiquiatr..

[43] Williams A.D., Bird M.-L., Hardcastle S.G., Kirschbaum M., Ogden K.J., Walters J.A. (2018). Exercise for Reducing Falls in People Living with and beyond Cancer. Cochrane Database Syst. Rev..

[44] Corcuera-Ciudad R., Patiño-Villena A.F., Paima-Olivari R., Chambergo-Michilot D., Parodi J.F., Runzer-Colmenares F.M. (2019). Trastornos de la marcha y el equilibrio en adultos mayores y su asociación con diabetes mellitus tipo 2. Med. Interna México.

[45] Liu-Ambrose T., Davis J.C., Best J.R., Dian L., Madden K., Cook W., Hsu C.L., Khan K.M. (2019). Effect of a Home-Based Exercise Program on subsequent Falls Among Community-Dwelling High-Risk Older Adults After a Fall: A Randomized Clinical Trial. JAMA.

[46] Baptista L.C., Amorim A.P., Valente-dos-Santos J., Machado-Rodrigues A.M., Veríssimo M.T., Martins R.A. (2018). Functional Status Improves in Hypertensive Older Adults: The Long-Term Effects of Antihypertensive Therapy Combined with Multicomponent Exercise Intervention. Aging Clin. Exp. Res..

[47] de Hoyos Alonso M.d.C., Gorroñogoitia Iturbe A., Martín Lesende I., Baena Díez J.M., López-Torres Hidalgo J., Magán Tapia P., Acosta Benito M.Á., Herreros Herreros Y. (2018). Actividades preventivas en los mayores. Actualización PAPPS 2018. Aten. Primaria.

